# Prejudice

**Published:** 1864-04

**Authors:** 


**Prejudice.**—All men are apt to have a high conceit of their own understanding, and to be tenacious of the opinions they profess; and yet almost all men are guided by the understandings of others, not by their own, and may be said more truly to adopt than to beget their opinions. Nurses, parents, pedagogues, and after them all, and above them all, that universal pedagogue system fills the mind with notions which it has no share in framing; which it receives as passively as it receives the impressions of outward objects, and which, left to itself, it would never have framed, or would have examined afterwards. Thus prejudices are established by education, and habits by custom. We are taught to think what others think, not how to think for ourselves; and whilst the memory is loaded, the understanding remains unexercised, or exercised in such trammels as constrain its motions and direct its pace, till that which is artificial becomes in some sort natural, and the mind can go to no other. It may sound oddly, but it is true in many cases, to say, that if men had learned less, their way to knowledge would be shorter and easier. It is, indeed, shorter and easier to proceed from ignorance to knowledge than from error. They who are in the last must unlearn before they can learn to any good purpose; and the first part of this double task is not in many respects the least difficult, for which reason it is seldom undertaken.—*Literary Journal.*

